# Supplementary material for: Complexome profiling of the Chlamydomonas psb28 mutant reveals TEF5 as an early PSII assembly factor
Source: Plant Cell. 2025 Mar 17;37(6):koaf055. doi: 10.1093/plcell/koaf055 (PMC12164590; doi:10.1093/plcell/koaf055)
Supplement: koaf055_Supplementary_Data [file koaf055_supplementary_data.zip › psb28_interactive.zip!/plots/intensity/qualityPlots/peptides/GDH1_Cre09.g388800.t1.2.html]

### Cre09.g388800.t1.2

Trivial name: GDH1  
SeqCoverage [median]: 15.00  
Mass: 48.56 kDa  
  
 

| Sequence | MissedCleavage | Mass [Da] | wtIntensity | muIntensity | Score |
| --- | --- | --- | --- | --- | --- |
| EALDLLDFDPQVEK | 0 | 1630.81 | 514.32 | 0.00 | 148.28 |
| GGIIYHPGVTLENMR | 0 | 1655.85 | 0.00 | 841.16 | 47.53 |
| GGVGVDPR | 0 | 755.39 | 588.79 | 692.21 | 70.91 |
| LQQADSVR | 0 | 915.48 | 842.79 | 645.25 | 122.13 |
| TAGYLVALR | 0 | 962.55 | 0.00 | 778.76 | 65.04 |
| VAGTSFLIQGFGK | 0 | 1323.72 | 431.25 | 0.00 | 117.70 |
|  |  |  | 2377.2 | 2957.4 |  |
